# Relationship between body composition and the histology of non‐alcoholic fatty liver disease: a cross‐sectional study

**DOI:** 10.1186/s12876-021-01748-y

**Published:** 2021-04-13

**Authors:** Teruki Miyake, Masumi Miyazaki, Osamu Yoshida, Sayaka Kanzaki, Hironobu Nakaguchi, Yoshiko Nakamura, Takao Watanabe, Yasunori Yamamoto, Yohei Koizumi, Yoshio Tokumoto, Masashi Hirooka, Shinya Furukawa, Eiji Takeshita, Teru Kumagi, Yoshio Ikeda, Masanori Abe, Kumiko Toshimitsu, Bunzo Matsuura, Yoichi Hiasa

**Affiliations:** 1 Department of Gastroenterology and Metabology, Ehime University Graduate School of Medicine, 791-0295 Shitsukawa, Toon, Ehime Japan; 2 Department of Lifestyle-Related Medicine and Endocrinology, Ehime University Graduate School of Medicine, Ehime Shitsukawa, Toon, Japan; 3 Health Service Center, Ehime University, Ehime Bunkyo, Matsuyama, Japan; 4 Post Graduate Medical Education Center, Ehime University Graduate School of Medicine, Ehime Shitsukawa, Toon, Japan; 5 Nutrition Division, Ehime University Hospital, Shitsukawa, Toon, Ehime Japan

**Keywords:** Fat mass, Muscle mass, Non‐alcoholic steatohepatitis, Liver fibrosis, Visceral fat

## Abstract

**Background:**

Causes of non-alcoholic fatty liver disease and its progression include visceral fat accumulation and loss of muscle mass; however, which of the two phenomena is more critical is unclear. Therefore, we intended to examine the relationship between body composition and non-alcoholic fatty liver disease progression as indicated by fibrosis and the non-alcoholic fatty liver disease activity score.

**Methods:**

This cross-sectional study comprised 149 patients (55 men; age, 20–76 years) treated for non-alcoholic fatty liver disease between December 2010 and January 2020. Body composition measurements, histological examinations of liver samples, and comprehensive blood chemistry tests were performed. The relationship between body composition and non-alcoholic fatty liver disease histology findings was analyzed using the logistic regression model.

**Results:**

Fibrosis was significantly and inversely correlated with muscle mass and appendicular skeletal muscle mass and significantly and positively correlated with fat mass, fat mass/height squared, visceral fat area, and waist-hip ratio (P < 0.05). After adjustment for sex, blood chemistry measurements, and body composition indices, fibrosis remained associated with appendicular skeletal muscle mass, fat mass, fat mass/height squared, and visceral fat area (P < 0.05). Non-alcoholic fatty liver disease activity score ≥ 5 significantly correlated with fat mass and fat mass/height squared in a univariate but not multivariate analysis.

**Conclusions:**

Fibrosis in non-alcoholic fatty liver disease, an indicator of unfavorable long-term outcomes, is associated with more indices of fat mass than of those of muscle mass. Hence, fat mass should be controlled to prevent non-alcoholic fatty liver disease progression.

**Supplementary Information:**

The online version contains supplementary material available at 10.1186/s12876-021-01748-y.

## Background

Non-alcoholic fatty liver disease (NAFLD) is one of the most common hepatic diseases and a manifestation of metabolic syndrome [1, 2]. NAFLD is a risk factor for various metabolic and cardiovascular diseases, and it can also progress to cirrhosis and liver failure [2–4]. Therefore, clinicians need to understand the pathophysiology of NAFLD and closely monitor patients with NAFLD to prevent progression and serious complications of the disease.

The causes of NAFLD are various. Several studies associate NAFLD with fat accumulation and loss of muscle mass [5–14]. Lack of exercise and overeating increase the size of the fat mass, particularly the amount of visceral adipose tissue (VAT). VAT accumulation induces insulin resistance and exacerbates liver damage in NAFLD [5]. Loss of skeletal muscle mass (i.e., sarcopenia) is associated with diabetes, metabolic syndrome, and cardiovascular disease and is a risk factor for non-alcoholic steatohepatitis (NASH) and fibrosis (≥ F2) [6–14]. Although few studies have reported on the relationship between fat mass and muscle mass and NAFLD, our study has evaluated the parameters in more detail than that in the previous studies, and it is unclear which of these factors is more critical than the other.

Elucidating the relationship between body composition and NAFLD is important for identifying patients at high risk of NAFLD progression. We examined this relationship using fibrosis stage and NAFLD activity score (NAS) as indicators of NAFLD progression. We also determined whether muscle mass or fat mass was more involved in NAFLD pathology.

## Methods

### Participants

We enrolled 157 patients with NAFLD (58 men and 99 women), who were aged 20–76 years and were treated from December 2010 to January 2021 at a single hospital. Patients were eligible for the study if they were diagnosed with fatty liver via liver biopsy and had either elevated liver enzyme levels or imaging (ultrasonography or computed tomography) findings indicative of possible liver injury, their body composition was evaluated within 3 days before and after the liver biopsy, they had no liver diseases of other etiologies, they consumed < 30 g/day (men) or < 20 g/day (women) of alcohol, and had no evidence of decompensated liver failure or hepatocellular carcinoma. Patient datasets were numerically coded to preserve anonymity, and data were housed in a secure database. Eight patients were excluded because either their data were missing, or they used steroids, had cancer, and/or were otherwise deemed inappropriate for this study (n = 2, 2, 3 and 1, respectively). Finally, 149 patients (55 men and 94 women) were included in the study, and their medical records were analyzed.

 This cross-sectional study was conducted in accordance with the ethical guidelines of the 1975 Declaration of Helsinki as revised in 1983 and was approved by the Ethics Committee of Ehime University Hospital (approval ID number: 1,012,004, 1,709,008; University Hospital Medical Information Network ID: UMIN000010659, UMIN 000030222). All study participants provided informed consent.

### Patient evaluation

Results of physical and biochemical examinations were assessed. Fasting venous blood samples were taken on the morning of the second day of hospitalization. Body composition, including skeletal muscle mass and body fat mass, was determined using an InBody720 analyzer (Biospace Corporation Limited, Seoul, Korea), with the patients wearing light gowns and no shoes. For biochemical analysis, the levels of alanine aminotransferase (ALT), γ-glutamyl transpeptidase (GGT), creatinine (Cre), hemoglobin A1c (HbA1c), total cholesterol (TC), and triglyceride (TG) were measured.

## Histological assessment

All patients in this study underwent percutaneous liver biopsy assisted by ultrasonography or laparoscopy. The liver specimens were embedded in paraffin and stained with hematoxylin & eosin and reticulin silver. Two experienced hepatopathologists who were blinded to the clinical features examined the liver biopsy specimens.

The NAS, which is the sum of the scores for steatosis (grade 0–3), lobular inflammation (grade 0–3), and ballooning degeneration (grade 0–2) [15], was recorded for each patient. Patients with an NAS ≥ 5 were diagnosed with NASH. Hepatic fibrosis was staged as described in previous reports: stage 0, absence of fibrosis; stage 1a, delicate perisinusoidal fibrosis; stage 1b, dense perisinusoidal fibrosis; stage 1c, portal-only fibrosis without perisinusoidal fibrosis; stage 2, combined perisinusoidal and portal/periportal fibrosis; stage 3, bridging fibrosis; and stage 4, cirrhosis [16].

### Statistical analysis

The Wilcoxon test, Kruskal-Wallis test, unpaired t-test, one-way analysis of variance, and logistic regression analysis were performed using JMP software (version 14.2; SAS Institute, Cary, NC, USA). Odds ratio (OR) and their 95 % confidence interval (CI) were determined to assess the relationship between the histological features of NAFLD and the indices of body composition. Factors that were significant in a univariate analysis were included in a multivariate analysis that was adjusted for the following potential confounding factors: age of patient; sex of patient; ALT, Cre, HbA1c, TC, and TG levels; and body composition indices, namely, muscle mass, muscle mass divided by height squared (ht^2^), fat mass, or fat mass divided by ht^2^. The cutoff levels of significant factors were assessed by analyzing the receiver operating characteristic (ROC) curve. Diagnostic accuracy was calculated by sensitivity, specificity, positive, and negative predictive values. Spearman’s correlation coefficients were used to investigate the relationship between body composition and HbA1c levels. Data are expressed as median (interquartile range) or number (percentage). A P-value of < 0.05 was considered statistically significant.

## Results

### Patient characteristics

Table 1 shows the patients’ characteristics. The median muscle mass, median muscle mass/ht^2^, appendicular skeletal muscle mass (ASM), ASM/ht^2^ (SMI: skeletal muscle index), fat mass, fat mass/ht^2^ (BFMI: body fat mass index), visceral fat area, waist-hip ratio, and SMI/BFMI were 23.4 kg (range 20–29.7 kg), 9.6 kg/m^2^ (range, 8.6–10.6 kg/m^2^), 17.7 kg (range, 14.4–22 kg), 7.1 kg/m^2^ (range, 6.2–7.9 kg/m^2^), 24.5 kg (range, 19.1–31.9 kg), 10.1 kg/m^2^ (range, 7.5–12.5 kg/m^2^), 129.2 cm^2^ (range, 111–155.1 cm^2^), 0.96 (range 0.92–1.00), and 0.71 (range 0.54–0.92), respectively.

**Table 1:** Patient characteristics

Variable	Median (IQR)
Age, years	59 (46–67)
Sex, n (male/female)	55/94
Body mass index, kg/m^2^	27.7 (24.8–31)
Alanine aminotransferase, U/L	64 (36.5–102)
γ-Glutamyl transpeptidase, U/L	61 (35.5–110.5)
Creatinine, µmol/L	58.3 (48.6–74.3)
Hemoglobin A1c, %	6.3 (5.7–7.2)
Total cholesterol, mmol/L	4.9 (4.2–5.6)
Triglyceride, mmol/L	1.5 (1.1–2.0)
Muscle mass, kg	23.4 (20–29.7)
Muscle mass/ht^2^, kg/m^2^	9.6 (8.6–10.6)
ASM, kg	17.7 (14.4–22)
SMI, kg/m^2^	7.1 (6.2–7.9)
Muscle mass (upper extremity), kg	4.7 (3.9–6.2)
Muscle mass (upper extremity)/ht^2^ kg/m^2^	1.9 (1.7–2.2)
Muscle mass of lower extremity, kg	13.1 (10.4–15.8)
Muscle mass of lower extremity/ht^2^ kg/m^2^	5.2 (4.6–5.8)
Fat mass, kg	24.5 (19.1–31.9)
BFMI, kg/m^2^	10.1 (7.5–12.5)
Visceral fat area, cm^2^	129.2 (111–155.1)
Waist-hip ratio	0.96 (0.92–1.00)
SMI/BFMI	0.71 (0.54–0.92)

IQR, interquartile range; ht^2^, height squared; ASM, appendicular skeletal muscle mass; SMI, skeletal muscle index; BFMI, body fat mass index

#### Relationship between histological findings and body composition

Fat mass, Fat mass/ht^2^, visceral fat area, and the waist-hip ratio were significantly higher in patients with high-stage fibrosis than in patients without fibrosis (Table 2). SMI/BFMI in patients with stage 0 was higher than that in patients of other stages (Table 2). However, none of the muscle mass indices differed significantly according to the fibrosis stage (Table 2). Muscle mass/ht^2^, ASM, SMI, muscle mass of upper extremity, muscle mass of upper extremity/ht^2^, muscle mass of lower extremity, muscle mass of lower extremity/ht^2^, fat mass, and visceral fat area were significantly higher in patients with severe steatosis than in patients with mild steatosis (Additional file 1). Waist-hip ratio in patients with grade 33–66 steatosis was higher than that in patients of other grades (Additional file 1). Among different lobular inflammation or ballooning grades, there were no significant differences in body composition (Additional files 2 and 3). Fat mass and BFMI were significantly higher in patients with a high versus low NAS, whereas visceral fat area, waist-hip ratio, and all of the muscle mass indices were not related to the NAS (Table 3). SMI/BFMI in patients with high NAS was significantly lower than that in patients with low NAS (Table 3).

**Table 2:** Body composition according to the stage of fibrosis

Index	Median (IQR)			P-value
	Stage 0 (n = 29)	Stage 1–2 (n = 60)	Stage 3–4 (n = 60)	
Muscle mass, kg	27.4 (21.7–31.7)	22.9 (19.9–29.7)	23 (19.2–28.3)	0.09
Muscle mass/ht^2^, kg/m^2^	9.8 (9.3–11)	9.5 (8.3–10.6)	9.4 (8.4–10.5)	0.29
ASM, kg	20.9 (15.8–23.1)	16.9 (14.4–21.8)	17.3 (13.9–21.5)	0.13
SMI, kg/m^2^	7.3 (6.9–8.1)	7.1 (6.1–7.8)	7.1 (6–8.1)	0.61
Muscle mass of upper extremity, kg	5.4 (4.2–6.3)	4.7 (3.8–6.1)	4.6 (3.8–6.2)	0.27
Muscle mass of upper extremity/ht^2^, kg/m^2^	2 (1.8–2.2)	2 (1.6–2.2)	1.9 (1.6–2.2)	0.85
Muscle mass of lower extremity, kg	15.4 (11.9–16.7)	12.2 (10.4–15.8)	12.5 (10.2–15.4)	0.08
Muscle mass of lower extremity/ht^2^, kg/m^2^	5.5 (5.1–5.8)	5.2 (4.5–5.7)	5.1 (4.4–5.9)	0.5
Fat mass, kg	21.1 (13.6–25.6)	26.1 (20.6–31.8)	26.3 (19.4–33.8)	0.03
BFMI, kg/m^2^	8.4 (4.9–10.9)	10.2 (7.8–12.6)	10.3 (7.8–14)	0.01
Visceral fat area, cm^2^	113.1 (90.2–135.5)	127.7 (115.4–152.2)	138.4 (117–158.3)	< 0.01
Waist-hip ratio	0.92 (0.9–0.96)	0.96 (0.92–0.99)	0.99 (0.92–1.01)	< 0.01
SMI/BFMI	0.88 (0.65–1.54)	0.66 (0.53–0.85)	0.7 (0.52–0.86)	< 0.01

Kruskal-Wallis test or one-way analysis of variance was used. P < 0.05 was considered statistically significant

IQR, interquartile range; ht^2^, height squared; ASM, appendicular skeletal muscle mass; SMI, skeletal muscle index; BFMI, body fat mass index

**Table 3:** Body composition according to the non-alcoholic fatty liver disease activity score (NAS)

Index	Median (IQR)		P-value
	NAS 0–4 (n = 70)	NAS 5–8 (n = 79)	
Muscle mass, kg	23.7 (19.9–29.8)	23.2 (20-29.7)	0.98
Muscle mass/ht^2^, kg/m^2^	9.6 (8.6–10.5)	9.6 (8.5–10.7)	0.67
ASM, kg	17.8 (14.1–22)	17.6 (14.7–22.2)	0.8
SMI, kg/m^2^	7.1 (6.1–7.9)	7.1 (6.3–8.2)	0.44
Muscle mass of upper extremity, kg	4.7 (3.9–6.1)	4.7 (3.9–6.2)	0.33
Muscle mass of upper extremity/ht^2^, kg/m^2^	1.9 (1.7–2.2)	2 (1.7–2.3)	0.43
Muscle mass of lower extremity, kg	13.3 (10.3–15.8)	13 (10.9–15.9)	0.83
Muscle mass of lower extremity/ht^2^, kg/m^2^	5.2 (4.5–5.7)	5.2 (4.6-6)	0.51
Fat mass, kg	22.5 (15.4–29)	26.5 (21.3–33.9)	< 0.01
BFMI, kg/m^2^	9.1 (6.4–11.6)	10.6 (8.6–13.2)	< 0.01
Visceral fat area, cm^2^	124.8 (104.7-149.2)	132.8 (117.7-156.8)	0.08
Waist-hip ratio	0.95 (0.91–0.99)	0.96 (0.92-1)	0.21
SMI/BFMI	0.79 (0.58–1.05)	0.66 (0.52–0.85)	< 0.01

Wilcoxon test or unpaired t-test was used. P < 0.05 was considered statistically significant

IQR, interquartile range; ht^2^, height squared; ASM, appendicular skeletal muscle mass; SMI, skeletal muscle index; BFMI, body fat mass index

#### Fat mass indices are more strongly associated with fibrosis than are muscle mass indices

In the univariate analysis, fibrosis (≥ Stage 1) was significantly correlated with muscle mass (OR: 0.94, 95 % CI 0.88–0.999), fat mass (OR: 1.08, 95 % CI 1.02–1.14), BFMI (OR: 1.25, 95 % CI 1.1–1.45), visceral fat area (OR: 1.02, 95 % CI 1.01–1.04), waist-hip ratio (OR: 3.02 × 10^5^, 95 % CI 1.55 × 10^2^–1.43 × 10^9^), and SMI/BFMI (OR: 0.1, 95 % CI 0.03–0.3) (Table 4). Multivariate analysis adjusted using Model 1: age (years) of patient, sex of patient, ALT and Cre levels, body composition; Model 2: age (years) of patient, sex of patient, HbA1c level, body composition; and Model 3: age (years) of patient, sex of patient, TC level, TG level, and body composition showed that fat mass, BFMI, visceral fat area, waist-hip ratio, SMI/BFMI remained significant [fat mass: adjusted OR: 1.09, 95 % CI 1.02–1.18 (Model 1), adjusted OR: 1.09, 95 % CI 1.02–1.18 (Model 2), adjusted OR: 1.1, 95 % CI 1.02–1.18 (Model 3); BFMI: adjusted OR: 1.25, 95 % CI 1.03–1.55 (Model 1), adjusted OR: 1.27, 95 % CI 1.06–1.55 (Model 2), adjusted OR: 1.27, 95 % CI 1.06–1.56 (Model 3); visceral fat area: adjusted OR: 1.02, 95 % CI 1.002–1.04 (Model 1), adjusted OR: 1.02, 95 % CI 1.01–1.05 (Model 2), adjusted OR: 1.02, 95 % CI 1.01–1.05 (Model 3); waist-hip ratio: adjusted OR: 2.97 × 10^5^, 95 % CI 1.64–1.52 × 10^9^ (Model 1), adjusted OR: 1.09 × 10^5^, 95 % CI 5.21–4.38 × 10^9^ (Model 2); SMI/BFMI: adjusted OR: 0.14, 95 % CI 0.02–0.63 (Model 1), adjusted OR: 0.13, 95 % CI 0.02–0.57 (Model 2), adjusted OR: 0.12, 95 % CI 0.02–0.55 (Model 3)] (Table 5). In the univariate analysis, having an NAS ≥ 5, which is indicative of NASH, was significantly associated with fat mass (OR: 1.07, 95 % CI 1.03–1.12), BFMI (OR: 1.18, 95 % CI 1.07–1.31), and SMI/BFMI (OR: 0.19, 95 % CI 0.06–0.53) (Table 6). Multivariate analysis adjusted using Model 2 and Model 3 showed that SMI/ BFMI remained significant [adjusted OR: 0.27, 95 % CI: 0.06–0.94 (Model 2), adjusted OR: 0.27, 95 % CI: 0.06–0.97 (Model 3)] (Table 7).

**Table 4:** Association of body composition with fibrosis (≥ Stage 1) by univariate analysis

Index	OR (95 % CI)	P-value
Muscle mass, kg	0.94 (0.88–0.999)	0.048
Muscle mass/ht^2^, kg/m^2^	0.83 (0.63–1.08)	0.16
ASM, kg	0.93 (0.86–1.01)	0.09
SMI, kg/m^2^	0.85 (0.61–1.18)	0.33
Fat mass, kg	1.08 (1.02–1.14)	< 0.01
BFMI, kg/m^2^	1.25 (1.1–1.45)	< 0.01
Visceral fat area, cm^2^	1.02 (1.01–1.04)	< 0.01
Waist-hip ratio	3.02 × 10^5^ (1.55 × 10^2^–1.43 × 10^9^)	< 0.01
SMI/BFMI	0.1 (0.03–0.3)	< 0.01

OR, odds ratio; CI, confidence interval; ht^2^, height squared; ASM, appendicular skeletal muscle mass; SMI, skeletal muscle index; BFMI, body fat mass index

**Table 5:** Association of body composition with fibrosis (≥ Stage 1) by multivariate analysis

Index	Model 1		Model 2		Model 3	
	OR (95 % CI)	P-value	OR (95 % CI)	P-value	OR (95 % CI)	P-value
Muscle mass, kg	0.97 (0.84–1.15)	0.79^a^	0.9 (0.77–1.05)	0.18^a^	0.92 (0.79–1.07)	0.28^a^
Fat mass, kg	1.09 (1.02–1.18)	0.02^b^	1.09 (1.02–1.18)	0.01^b^	1.1 (1.02–1.18)	0.01^b^
BFMI, kg/m^2^	1.25 (1.03–1.55)	0.02^c^	1.27 (1.06–1.55)	0.02^c^	1.27 (1.06–1.56)	0.02^c^
Visceral fat area, cm^2^	1.02 (1.002–1.04)	0.03^b^	1.02 (1.01–1.05)	< 0.01 ^b^	1.02 (1.01–1.05)	< 0.01^b^
Waist-hip ratio	2.97 × 10^5^ (1.64–1.52 × 10^9^)	0.04^c^	1.09 × 10^5^ (5.21–4.38 × 10^9^)	0.02^c^	9.31 × 10^4^ (0.78–3.68 × 10^8^)	0.07^c^
SMI/BFMI	0.14 (0.02–0.63)	0.02	0.13 (0.02–0.57)	< 0.01	0.12 (0.02–0.55)	< 0.01

Model 1 was adjusted for age (years) of patient and sex of patient, and alanine aminotransferase (U/L) and creatinine (µmol/L) levels, along with ^a^fat mass (kg), ^b^muscle mass (kg), and ^c^muscle mass/ht^2^ (kg/m^2^)

Model 2 was adjusted for age (years) of patient, sex of patient, and hemoglobin A1c levels (%), along with ^a^fat mass (kg), ^b^muscle mass (kg), and ^c^muscle mass/ht^2^ (kg/m^2^)

Model 3 was adjusted for age (years) of patient and sex of patient, and total cholesterol (mmol/L) and triglyceride levels (mmol/L), along with ^a^fat mass (kg), ^b^muscle mass (kg), and ^c^muscle mass/ht^2^ (kg/m^2^)

OR, odds ratio; CI, confidence interval; BFMI, body fat mass index; SMI, skeletal muscle index; ht^2^, height squared

**Table 6:** Association between body composition and a non-alcoholic fatty liver disease activity score ≥ 5 by univariate analysis

Index	OR (95 % CI)	P-value
Muscle mass, kg	1.01 (0.95–1.06)	0.84
Muscle mass/ht^2^, kg/m^2^	1.08 (0.87–1.34)	0.5
ASM, kg	1.01 (0.94–1.08)	0.79
SMI, kg/m^2^	1.11 (0.86–1.45)	0.43
Fat mass, kg	1.07 (1.03–1.12)	< 0.01
BFMI, kg/m^2^	1.18 (1.07–1.31)	< 0.01
Visceral fat area, cm^2^	1.01 (0.999–1.02)	0.08
Waist-hip ratio	35.29 (0.22–7.42 × 10^3^)	0.17
SMI/BFMI	0.19 (0.06–0.53)	< 0.01

OR, odds ratio; CI, confidence interval; ht^2^, height squared; ASM, appendicular skeletal muscle mass; SMI, skeletal muscle index; BFMI, body fat mass index

**Table 7:** Association of body composition with non-alcoholic fatty liver disease activity score ≥ 5 by multivariate analysis

Index	Model 1		Model 2		Model 3	
	OR (95 % CI)	P-value	OR (95 % CI)	P-value	OR (95 % CI)	P-value
Fat mass, kg	1.04 (0.98–1.11)	0.2^a^	1.04 (0.99–1.1)	0.11^a^	1.04 (0.99–1.1)	0.16^a^
BFMI, kg/m^2^	1.1 (0.94–1.3)	0.23^b^	1.11 (0.97–1.27)	0.12^b^	1.1 (0.96–1.26)	0.19^b^
SMI/BFMI	0.27 (0.04–1.3)	0.13	0.27 (0.06–0.94)	0.04	0.27 (0.06–0.97)	0.04

Model 1 was adjusted for age (years) and sex of patient, and alanine aminotransferase (U/L) and creatinine (µmol/L) levels, along with ^a^muscle mass (kg) and ^b^muscle mass/ht^2^ (kg/m^2^)

Model 2 was adjusted for age(years) and sex of patient, and hemoglobin A1c levels (%), along with ^a^muscle mass (kg) and ^b^muscle mass/ht^2^ (kg/m^2^)

Model 3 was adjusted for age(years) and sex of patient, and total cholesterol and triglyceride levels, along with ^a^muscle mass (kg) and ^b^muscle mass/ht^2^ (kg/m^2^)

OR, odds ratio; CI, confidence interval; BFMI, body fat mass index; SMI, skeletal muscle index; ht^2^, height squared

#### The cutoff value of fat mass indices and SMI/BFMI for fibrosis

Among the significant risk factors for fibrosis, fat mass, BFMI, visceral fat area, and SMI/BFMI were selected for estimation of their cutoff level in diagnosing fibrosis. As shown in Table 8, the area under the ROC curve, cutoff level, sensitivity, specificity, positive predictive value, negative predictive value, and diagnostic accuracy in predicting fibrosis were follows: fat mass: 0.66, 26.3 kg, 50 %, 79.3 %, 90.9 %, 27.7 %, and 55.7 %; fat mass/ht^2^: 0.68, 7.44 kg/m^2^, 81.7 %, 44.8 %, 86 %, 37.1 %, and 74.5 %; visceral fat area: 0.67, 113.9 cm^2^, 78.3 %, 51.7 %, 86.2 %, 36.6 %, and 73.1 %, SMI/BFMI: 0.71, 0.87, 78.3 %, 55.2 %, 87.9 %, 38.1 %, and 73.8 %, respectively.

**Table 8:** Results of receiver operating characteristic curve analysis for fibrosis

	AU(ROC)	Cutoff level	Sensitivity (%)	Specificity (%)	PPV (%)	NPV (%)	Diagnostic accuracy (%)	P-value
Fat mass (kg)	0.66	26.3	50	79.3	90.9	27.7	55.7	< 0.01
BFMI (kg/m^2^)	0.68	7.44	81.7	44.8	86	37.1	74.5	< 0.01
Visceral fat area (cm^2^)	0.67	113.9	78.3	51.7	86.2	36.6	73.1	< 0.01
SMI/BFMI	0.71	0.87	78.3	55.2	87.9	38.1	73.8	< 0.01

AU(ROC), area under receiver operating characteristic curve; PPV, positive predictive value; NPV, negative predictive value; BFMI, body fat mass index; SMI, skeletal muscle index

#### The relationship between fat mass indices and HbA1c level

In patients without fibrosis (Stage 0), HbA1c level was not correlated with fat mass and fat/ht^2^ (r = 0.07, p = 0.72 and r = 0.07, p = 0.72, respectively.). However, in patients with fibrosis (≥ Stage 1), HbA1c level was correlated with fat mass and fat/ht^2^ (r = 0.23, p < 0.01 and r = 0.22, p = 0.01, respectively).

## Discussion

In this cross-sectional cohort study, we examined the relationship between the histological progression of NAFLD and body composition. We found that fibrosis in NAFLD was associated with more fat mass indices than muscle mass indices. Furthermore, our results remained significant after adjusting for possible confounders. Our findings suggest that the pathophysiology of NAFLD may be more dependent on fat accumulation than on loss of muscle mass. Hence, fat mass should be controlled to prevent the progression of NAFLD and avoid serious complications such as liver failure and hepatocellular carcinoma.

The effect of visceral fat deposition on the pathology of NAFLD has been reported previously [17]. Over-nutrition increases the size of the fat mass and accumulated fat, particularly the VAT supplies fatty acids to the liver. When in excess, fatty acids exacerbate steatosis [18–20], worsen lipid metabolism, generate reactive oxygen species, and injure the liver [21–23]. Additionally, disturbed adipocytokines in the accumulated VAT promote hepatic steatosis [24–26] and the production of proinflammatory macrophages [27, 28] and are associated with the development of NASH. Subcutaneous adipose tissue (SAT) also affects the pathogenesis of NAFLD. The number of macrophages in the SAT correlates with the amount of liver fat [29], and macrophage infiltration is significantly elevated in the deep, but not in the superficial, SAT in obese patients with NASH [30]. The expression of gene products that regulate inflammation in the SAT also correlates with the amount of liver fat, as well as with the histological features of NAFLD. Gene expression patterns in both the VAT and SAT suggest that these tissues promote the pathological progression of NAFLD through similar mechanisms [31].

Contrarily, although HbA1c level in patients without fibrosis (Stage 0) was not correlated with fat mass and fat/ht^2^, HbA1c level in patients with fibrosis (≥ Stage 1) was weakly correlated with fat mass and fat/ht^2^. These results suggest that accumulation of fat in the body strongly affects metabolic diseases in the presence of liver damage. The reason for a significant association between indices of fat mass and fibrosis but not NAS including inflammation may be that fibrosis reflects a long-term effect, whereas inflammation reflects a relatively short-term effect. Therefore, it seems that there was a stronger relationship with fibrosis than with NAS.

In our study, muscle mass was unrelated to the histological severity of NAFLD. The skeletal muscle index (skeletal muscle mass divided by height squared or weight) in patients with NAFLD was lower than that in healthy subjects [7]. Lee et al. examined three cohort studies, which consisted of 6567, 4587, and 4236 participants, respectively [8]. They defined low skeletal muscle mass index as appendicular skeletal muscle mass/BMI < 0.789 in men and < 0.512 in women or as the lowest quintile of total skeletal muscle mass/BMI by sex and showed that low skeletal muscle mass index was the risk factor for onset of NAFLD [8]. Kim et al. examined 12,624 subjects without baseline NAFLD and 2,943 subjects with baseline NAFLD [9] and showed that increased SMI, defined as appendicular skeletal muscle mass/weight, was associated with reduced incidence of NAFLD, and participants in the highest tertile of change in SMI over 1 year were associated with both a lower incident rate of NAFLD and a higher resolution rate of baseline NAFLD than those in the lowest tertile [9]. Additionally, Shi et al. enrolled 3255 subjects and showed that visceral fat area to appendicular muscle mass ratio (VAR) is a risk factor for NAFLD in men and women. They also calculated the suitable cutoff VAR values as 3.469 and 6.357 for men and women, respectively [10]. Additionally, after control of the influence of obesity, individuals with VAR above the cutoff value had a significantly higher risk of NAFLD [10]. However, the subjects of these studies have not been histologically examined and the relationship between NAFLD progression and body composition is unknown.

In another study of 123 patients with biopsy-confirmed NASH and 117 patients with biopsy-confirmed non-alcoholic fatty liver, sarcopenia (defined as an ASM/body weight value two standard deviations below the average for healthy young adults) significantly correlated with significant fibrosis (≥ stage F2) and NASH after adjusting for obesity, metabolic factors, and insulin resistance [11]. In another report of 225 patients with NAFLD diagnosed via liver biopsy, sarcopenia (defined as an ASM/body weight value ≤ 37 in men and ≤ 28 in women) significantly correlated with the severity of fibrosis and steatosis after adjustment for metabolic risk factors [12]. Moreover, Hsieh et al. conducted a cross-sectional cohort study with 521 biopsy-confirmed patients having NAFLD, and examined the relationship of low skeletal muscle mass (the height-adjusted skeletal muscle area [cm^2^/m^2^] < 50 cm^2^/m^2^ for men and < 39 cm^2^/m^2^ for women), myosteatosis (< 42.57 HU [Hounsfield unit] in patients with BMI ≥ 25 and < 39.77 HU in patients with BMI < 25), and visceral adiposity (the height-adjusted visceral adipose area [cm^2^/m^2^] > 60 cm^2^/m^2^ for men and > 68.23 cm^2^/m^2^ for women) using computed tomography for assessing the third lumbar vertebra and fibrosis severity [13]. They showed that low skeletal muscle mass, myosteatosis, and visceral adiposity were independent predictors of significant fibrosis (≥ stage F2) [13].

In contrast, a review of 136 patients with NASH and 129 patients with alcoholic liver disease found no association between sarcopenia (defined as an L3 skeletal muscle area/height/height value < 50 in men and < 39 in women) and poor wait-list outcomes, such as increased delisting risk and poorer wait-list survival [32]. Our study, unlike the aforementioned studies, did not include a cutoff value. Further investigation is required to clarify the association between muscle mass and NAFLD. However, a study by Alferink et al. provided partial support for our results [33]. The investigators examined data from the 4609 participants of the Rotterdam study, a population-based study in the Netherlands that evaluated body composition using dual-energy X-ray absorptiometry scanning, hepatic steatosis using abdominal ultrasonography, liver stiffness using transient elastography, grip strength using a hydraulic hand dynamometer, and gait speed using the GAITRite walkway [33]. The participants were stratified by sex and BMI, and the results demonstrated that high fat mass and fat distribution were more strongly associated with the high prevalence of NAFLD than was low muscle mass, while the high prevalence of presarcopenia and sarcopenia was not associated with high prevalence of NAFLD [33]. In normal-weight women, higher muscle mass was associated with a lower prevalence of both NAFLD and liver stiffness. However, histological findings were not examined [33]. Additionally, Mizuno et al. have examined the effect of skeletal muscle mass and body fat mass on liver function in patients with NAFLD, who were diagnosed by liver biopsy, and showed that the body fat mass index (BFMI) (kg/m^2^), but not skeletal muscle index (SMI) (kg/m^2^), was significantly higher in patients with NASH than in those with NAFL [34]. Moreover, changes in the SMI/BFMI were significantly associated with changes in liver enzyme, independent of age and other backgrounds [34]. However, our study focused on the various indices of skeletal muscle mass and fat mass and examined the relationship between histological findings and various indices of skeletal muscle mass and fat mass in more detail than that in Mizuno et al.’s study to compare whether muscle or fat is strongly associated with the pathology of NAFLD.

The strengths of our study are that patients were diagnosed with NAFLD via liver biopsy and that the examination of whole-body fat mass and muscle mass was conducted at the same facility under the same conditions. However, our study also had several limitations. First, our study participants were all Japanese. Body composition differs among races; therefore, whether our results can be generalized to other races is uncertain. Second, we did not measure skeletal muscle strength and function [35] and did not examine the relationship between NAFLD histology and sarcopenia. However, our aim for this study was to examine the relationship between body composition and NAFLD histology. Third, the total number of cases is small. Fourth, we did not consider the BMI of the patients included in this study while analyzing the body composition. This was because there was no significant difference in the proportion of patients with NASH among the subjects when they were separated by BMI (≥ 25 and < 25 [83.3 % vs. 73.1 %]), and our method of measuring body composition was unaffected by obesity. Finally, because our study design was cross-sectional a causal relationship between NAFLD and body composition could not be established. Therefore, whether patients with an enlarged fat mass are at high risk for NAFLD progression remains unknown. Future validation studies are necessary to address these limitations.

## Conclusions

Despite its limitations, our study had several notable results. Particularly, it associates fibrosis, an indicator of unfavorable long-term outcomes, with indices of fat accumulation in patients with NAFLD and suggests that fat mass more strongly impacts the pathophysiology of NASH than muscle mass does. Therefore, correct recognition is critical for identifying patients at high risk of NAFLD progression.

## Supplementary Information

**Additional file 1:** Body composition according to steatosis grade.

**Additional file 2:** Body composition according to lobular inflammation grade.

**Additional file 3:** Body composition according to ballooning grade.

## Data Availability

The datasets used and/or analyzed during the current study are available from the corresponding author on reasonable request. All authors have read and agreed to the published version of the manuscript.

## Abbreviations

ALT: Alanine aminotransferase
ASM: Appendicular skeletal muscle mass
BFMI: Body fat mass index
CI: Confidence interval
Cre: Creatinine
GGT: γ-glutamyl transpeptidase
HbA1c: Hemoglobin A1c
ht2: Height squared
HU: Hounsfield unit
NAFLD: Non-alcoholic fatty liver disease
NAS: NAFLD activity score
NASH: Non-alcoholic steatohepatitis
OR: Odds ratio
ROC: Receiver operating characteristic
SAT: Subcutaneous adipose tissue
SMI: Skeletal muscle index
TC: total cholesterol
TG: Triglyceride
VAR: Visceral fat area to appendicular muscle mass ratio
VAT: Visceral adipose tissue
