# Supplementary material for: Relationship between body composition and the histology of non‐alcoholic fatty liver disease: a cross‐sectional study
Source: BMC Gastroenterol. 2021 Apr 13;21:170. doi: 10.1186/s12876-021-01748-y (PMC8045325; doi:10.1186/s12876-021-01748-y)
Supplement: Supplementary file 1 — Additional file 1: Body composition according to steatosis grade. [file 12876_2021_1748_MOESM1_ESM.docx]

**Relationship between body composition and the histology of non-alcoholic fatty liver disease: a cross-sectional study**

Teruki Miyake^1^, Masumi Miyazaki^1^, Osamu Yoshida^1^, Sayaka Kanzaki^1^, Hironobu Nakaguchi^2^, Yoshiko Nakamura^1^, Takao Watanabe^1^, Yasunori Yamamoto^1^, Yohei Koizumi^1^, Yoshio Tokumoto^1^, Masashi Hirooka^1^, Shinya Furukawa^3^, Eiji Takeshita^1^, Teru Kumagi^4^, Yoshio Ikeda^1^, Masanori Abe^1^, Kumiko Toshimitsu^5^, Bunzo Matsuura^2^, Yoichi Hiasa^1^

^1^Department of Gastroenterology and Metabology, Ehime University Graduate School of Medicine, Shitsukawa, Toon, Ehime, Japan

^2^Department of Lifestyle-related Medicine and Endocrinology, Ehime University Graduate School of Medicine, Shitsukawa, Toon, Ehime, Japan

^3^Health service center, Ehime University, Bunkyo, Matsuyama, Ehime, Japan

^4^Post graduate medical education center, Ehime University Graduate School of Medicine, Shitsukawa, Toon, Ehime, Japan

^5^Nutrition Division, Ehime University Hospital, Shitsukawa, Toon, Ehime, Japan

Corresponding author:

Yoichi Hiasa, M.D., Ph.D.

Department of Gastroenterology and Metabology

Ehime University Graduate School of Medicine

Toon, Ehime 791-0295, Japan

Phone: +81 89 960 5308

Fax: +81 89 960 5310

E-mail: [hiasa@m.ehime-u.ac.jp](mailto:hiasa@m.ehime-u.ac.jp)

**Additional file 1**

**Body composition according to steatosis grade**

| Index | Median (IQR) | | | P-value |
| --- | --- | --- | --- | --- |
|  | Grade <33  (n = 52) | Grade 33–66  (n = 50) | Grade >66  (n = 47) |  |
| Muscle mass, kg | 21.4 (19.2–27.3) | 24.8 (19.9–30) | 24.1 (20.5–32.3) | 0.05 |
| Muscle mass/ht^2^, kg/m^2^ | 9.2 (8.2–10.3) | 9.7 (8.8–10.7) | 9.8 (9–11.3) | 0.03 |
| ASM, kg | 15.5 (13.7–20.5) | 18.9 (14.7–22.3) | 18 (15.2–23.7) | 0.02 |
| SMI, kg/m^2^ | 6.7 (5.8–7.6) | 7.3 (6.6–8.2) | 7.3 (6.5–8.3) | <0.01 |
| Muscle mass of upper extremity, kg | 4.4 (3.6–5.4) | 5 (4–6.3) | 4.9 (4.1–6.6) | 0.04 |
| Muscle mass of upper extremity/ht^2^, kg/m^2^ | 1.8 (1.6–2.1) | 2 (1.7–2.3) | 2 (1.7–2.3) | 0.01 |
| Muscle mass of lower extremity, kg | 11.6 (9.8–15.3) | 13.8 (10.7–15.9) | 13.2 (11.2–16.7) | 0.03 |
| Muscle mass of lower extremity/ht^2^, kg/m^2^ | 4.7 (4.4–5.6) | 5.2 (4.7–5.9) | 5.4 (4.8–6) | <0.01 |
| Fat mass, kg | 22.3 (15.3–29.7) | 24.6 (20.3–33.5) | 27.2 (21.3–34.9) | <0.01 |
| BFMI, kg/m^2^ | 9.6 (5.8–11.7) | 9.8 (7.8–13) | 10.7 (8.8–12.9) | 0.07 |
| Visceral fat area, cm^2^ | 121.9 (102.8–139) | 144.4 (121.5–159.8) | 126.6 (111.9–157) | 0.01 |
| Waist-hip ratio | 0.95 (0.91–1) | 0.97 (0.94–1) | 0.94 (0.91–0.98) | 0.04 |
| SMI/BFMI | 0.77 (0.52–1.14) | 0.7 (0.54–0.99) | 0.7 (0.57–0.84) | 0.52 |

Kruskal-Wallis test or one-way analysis of variance was used. P <0.05 was considered statistically significant.

IQR, interquartile range; ht^2^, height squared; ASM, appendicular skeletal muscle mass; SMI, skeletal muscle index; BFMI, body fat mass index
