# Supplementary material for: Relationship between body composition and the histology of non‐alcoholic fatty liver disease: a cross‐sectional study
Source: BMC Gastroenterol. 2021 Apr 13;21:170. doi: 10.1186/s12876-021-01748-y (PMC8045325; doi:10.1186/s12876-021-01748-y)
Supplement: Supplementary file 2 — Additional file 2: Body composition according to lobular inflammation grade. [file 12876_2021_1748_MOESM2_ESM.docx]

**Relationship between body composition and the histology of non-alcoholic fatty liver disease: a cross-sectional study**

Teruki Miyake^1^, Masumi Miyazaki^1^, Osamu Yoshida^1^, Sayaka Kanzaki^1^, Hironobu Nakaguchi^2^, Yoshiko Nakamura^1^, Takao Watanabe^1^, Yasunori Yamamoto^1^, Yohei Koizumi^1^, Yoshio Tokumoto^1^, Masashi Hirooka^1^, Shinya Furukawa^3^, Eiji Takeshita^1^, Teru Kumagi^4^, Yoshio Ikeda^1^, Masanori Abe^1^, Kumiko Toshimitsu^5^, Bunzo Matsuura^2^, Yoichi Hiasa^1^

^1^Department of Gastroenterology and Metabology, Ehime University Graduate School of Medicine, Shitsukawa, Toon, Ehime, Japan

^2^Department of Lifestyle-related Medicine and Endocrinology, Ehime University Graduate School of Medicine, Shitsukawa, Toon, Ehime, Japan

^3^Health service center, Ehime University, Bunkyo, Matsuyama, Ehime, Japan

^4^Post graduate medical education center, Ehime University Graduate School of Medicine, Shitsukawa, Toon, Ehime, Japan

^5^Nutrition Division, Ehime University Hospital, Shitsukawa, Toon, Ehime, Japan

Corresponding author:

Yoichi Hiasa, M.D., Ph.D.

Department of Gastroenterology and Metabology

Ehime University Graduate School of Medicine

Toon, Ehime 791-0295, Japan

Phone: +81 89 960 5308

Fax: +81 89 960 5310

E-mail: [hiasa@m.ehime-u.ac.jp](mailto:hiasa@m.ehime-u.ac.jp)

**Additional file 2**

**Body composition according to lobular inflammation grade**

| Index | Median (IQR) | | | P-value |
| --- | --- | --- | --- | --- |
|  | Grade <2 (n = 73) | Grade 2–4 (n = 53) | Grade ≥5 (n = 23) |  |
| Muscle mass, kg | 23.4 (20–30.1) | 22.8 (19.6–28.8) | 24.8 (20.2–33.4) | 0.2 |
| Muscle mass/ht^2^, kg/m^2^ | 9.6 (8.5–10.6) | 9.4 (8.5–10.6) | 9.8 (9–12) | 0.38 |
| ASM, kg | 18.5 (14.1–22.3) | 17.2 (14.3–21.7) | 18.2 (14.7–24.9) | 0.23 |
| SMI, kg/m^2^ | 7.2 (6.1–7.9) | 7 (6.1–7.9) | 7.2 (6.5–8.7) | 0.23 |
| Muscle mass of upper extremity, kg | 4.7 (3.9–6.2) | 4.7 (3.7–5.9) | 5.1 (4.2–7) | 0.31 |
| Muscle mass of upper extremity/ht^2^, kg/m^2^ | 1.9 (1.7–2.2) | 2 (1.6–2.2) | 2.1 (1.7–2.4) | 0.23 |
| Muscle mass of lower extremity, kg | 13.7 (10.3–15.9) | 12 (10.4–15.7) | 13.4 (11.2–17.9) | 0.26 |
| Muscle mass of lower extremity/ht^2^, kg/m^2^ | 5.2 (4.5–5.8) | 5.1 (4.5–5.7) | 5.3 (4.6–6.2) | 0.25 |
| Fat mass, kg | 24.4 (17.9–30.4) | 24.1 (19–32.9) | 28.9 (22.4–33) | 0.13 |
| BFMI, kg/m^2^ | 9.8 (7.1–11.7) | 10.2 (7.6–13.7) | 11.4 (8.6–12.9) | 0.15 |
| Visceral fat area, cm^2^ | 127.5 (109.2–155.5) | 125.2 (105.2–145.6) | 134.5 (123.8–160.5) | 0.22 |
| Waist-hip ratio | 0.95 (0.91–0.99) | 0.96 (0.92–1) | 0.96 (0.93–1) | 0.54 |
| SMI/BFMI | 1.75 (0.58–1.98) | 0.67 (0.51–0.86) | 0.74 (0.53–1) | 0.22 |

Kruskal-Wallis test or one-way analysis of variance was used. P <0.05 was considered statistically significant.

IQR, interquartile range; ht^2^, height squared; ASM, appendicular skeletal muscle mass; SMI, skeletal muscle index; BFMI, body fat mass index
