# Supplementary material for: Relationship between body composition and the histology of non‐alcoholic fatty liver disease: a cross‐sectional study
Source: BMC Gastroenterol. 2021 Apr 13;21:170. doi: 10.1186/s12876-021-01748-y (PMC8045325; doi:10.1186/s12876-021-01748-y)
Supplement: Supplementary file 3 — Additional file 3: Body composition according to ballooning grade. [file 12876_2021_1748_MOESM3_ESM.docx]

**Relationship between body composition and the histology of non-alcoholic fatty liver disease: a cross-sectional study**

Teruki Miyake^1^, Masumi Miyazaki^1^, Osamu Yoshida^1^, Sayaka Kanzaki^1^, Hironobu Nakaguchi^2^, Yoshiko Nakamura^1^, Takao Watanabe^1^, Yasunori Yamamoto^1^, Yohei Koizumi^1^, Yoshio Tokumoto^1^, Masashi Hirooka^1^, Shinya Furukawa^3^, Eiji Takeshita^1^, Teru Kumagi^4^, Yoshio Ikeda^1^, Masanori Abe^1^, Kumiko Toshimitsu^5^, Bunzo Matsuura^2^, Yoichi Hiasa^1^

^1^Department of Gastroenterology and Metabology, Ehime University Graduate School of Medicine, Shitsukawa, Toon, Ehime, Japan

^2^Department of Lifestyle-related Medicine and Endocrinology, Ehime University Graduate School of Medicine, Shitsukawa, Toon, Ehime, Japan

^3^Health service center, Ehime University, Bunkyo, Matsuyama, Ehime, Japan

^4^Post graduate medical education center, Ehime University Graduate School of Medicine, Shitsukawa, Toon, Ehime, Japan

^5^Nutrition Division, Ehime University Hospital, Shitsukawa, Toon, Ehime, Japan

Corresponding author:

Yoichi Hiasa, M.D., Ph.D.

Department of Gastroenterology and Metabology

Ehime University Graduate School of Medicine

Toon, Ehime 791-0295, Japan

Phone: +81 89 960 5308

Fax: +81 89 960 5310

E-mail: [hiasa@m.ehime-u.ac.jp](mailto:hiasa@m.ehime-u.ac.jp)

**Additional file 3**

**Body composition according to ballooning grade**

| Index | Median (IQR) | | | P-value |
| --- | --- | --- | --- | --- |
|  | Grade 0 (n = 28) | Grade 1 (n = 70) | Grade 2 (n = 51) |  |
| Muscle mass, kg | 27.6 (20.9–30.9) | 22.5 (19.9–28.5) | 23.4 (19.7–30) | 0.13 |
| Muscle mass/ht^2^, kg/m^2^ | 9.9 (9–11) | 9.3 (8.3–10.4) | 9.6 (8.5–10.6) | 0.21 |
| ASM, kg | 21.1 (14.7–23.8) | 17 (14.3–21.1) | 17.6 (14.2–22.2) | 0.22 |
| SMI, kg/m^2^ | 7.3 (6.7–8.2) | 7 (6–7.8) | 7.1 (6.3–8.2) | 0.34 |
| Muscle mass of upper extremity, kg | 5.3 (4–6.3) | 4.5 (3.9–5.9) | 4.7 (3.8–6.2) | 0.37 |
| Muscle mass of upper extremity/ht^2^, kg/m^2^ | 1.9 (1.7–2.2) | 1.9 (1.6–2.2) | 2 (1.7–2.3) | 0.55 |
| Muscle mass of lower extremity, kg | 15.6 (10.7–17.5) | 12.1 (10.4–15.4) | 12.9 (10.9–15.8) | 0.12 |
| Muscle mass of lower extremity/ht^2^, kg/m^2^ | 5.5 (4.9–5.9) | 5.1 (4.5–5.7) | 5.2 (4.7–6.0) | 0.19 |
| Fat mass, kg | 22.5 (15–28.4) | 24.6 (18.5–31.9) | 25.8 (20.1–33.9) | 0.2 |
| BFMI, kg/m^2^ | 9.2 (5.8–11.5) | 10.3 (7.5–12.8) | 10.3 (8.4–12.5) | 0.14 |
| Visceral fat area, cm^2^ | 122 (101.6–153.1) | 129.4 (107.7–156.2) | 134.5 (117.7–156.8) | 0.42 |
| Waist-hip ratio | 0.94 (0.90–0.98) | 0.96 (0.92–1) | 0.96 (0.92–1) | 0.1 |
| SMI/BFMI | 0.84 (0.63–1.12) | 0.69 (0.52–0.87) | 0.67 (0.54–0.87) | 0.06 |

Kruskal-Wallis test or one-way analysis of variance was used. P <0.05 was considered statistically significant.

IQR, interquartile range; ht^2^, height squared; ASM, appendicular skeletal muscle mass; SMI, skeletal muscle index; BFMI, body fat mass index
